# Verbal and Figural Creativity in Children With Autism Spectrum Disorder and Typical Development

**DOI:** 10.3389/fpsyg.2020.559238

**Published:** 2020-10-27

**Authors:** Anat Kasirer, Esther Adi-Japha, Nira Mashal

**Affiliations:** ^1^School of Education, Bar-Ilan University, Ramat Gan, Israel; ^2^Gonda Multidisciplinary Brain Research Center, Bar-Ilan University, Ramat Gan, Israel

**Keywords:** creativity, verbal creativity, ASD, drawing, figural, non-verbal creativity

## Abstract

Previous studies have shown that individuals with autism spectrum disorder (ASD) demonstrate lower performance on creativity tasks. Yet, recent findings suggest that individuals with ASD are not necessarily impaired in verbal creativity, as measured by the novel metaphor generation task. The current study investigates verbal and figural creativity in 40 children with ASD (aged 11–14 years) and 39 peers with typical development (TD) (aged 11–15 years). We also tested the contribution of executive functions to the creative performance. A sentence completion questionnaire was used to test creative verbal generation, while a task of drawing non-existent objects was used to assess figural abilities. The results indicate that children with ASD generated a greater quantity of creative metaphors and showed greater use of a specific kind of representational change on the figural creativity task: cross-category insertions (e.g., a house with a tail). However, no correlation was found between the metaphor generation task and the use of cross-category insertions for either group. Results also showed that, whereas phonemic fluency contributed to the explained variance in novel metaphor generation in the ASD group, fluid intelligence, although only marginally, contributed to variance in novel metaphor generation in the TD group. These findings suggest that verbal creativity and figural creativity are two separate abilities relying on different cognitive resources. Our results show that those with ASD and TD differ in the cognitive abilities they use to perform the metaphor generation task. The research points to a unique creative cognition profile among children with ASD.

## Introduction

Much has been written about the difficulties in figurative language comprehension among children with autism, and, in particular, their tendency to provide literal interpretations to metaphoric utterances (Happé, 1993, 1994; MacKay and Shaw, 2004; Rundblad and Annaz, 2010; Mashal and Kasirer, 2011; Kasirer and Mashal, 2014, 2016; Melogno et al., 2017; Kalandadze et al., 2018). Most of the studies using figurative language tasks in ASD focused on metaphor comprehension but very little is known about metaphor generation in ASD. The current study focuses on two generation tasks: verbal and non-verbal (figural) tasks, both of which may shed light on creative abilities in ASD.

The difficulties in figurative language comprehension among those with ASD are supported by a recent meta-analysis that showed poorer comprehension of figurative language among individuals with autism spectrum disorder (ASD) compared to individuals with typical development (TD) matched on chronological age or/and language ability (Kalandadze et al., 2018). Although evidence suggests that that there is a pervasive problem in ASD in figurative language comprehension, there is still a debate concerning the causes of this deficit and what aspects of the autistic profile account for this difficulty. According to Kalandadze et al.’s (2018) meta-analysis, when individuals with ASD and TD are compared based on their performance on core language tests (vocabulary, syntax), both groups demonstrate similar performance in figurative language. However, in studies that included chronological age (CA) matching, group differences were large, and in studies that included both CA- and language ability matching, the group differences were moderate. It has been shown that core language skills predict figurative language comprehension in individuals with ASD even after ToM ability is controlled (Norbury, 2005). Nevertheless, not many studies tested ToM ability alongside language skills in individuals with ASD; thus, the extent to which language skills and ToM independently contribute to figurative language comprehension remains to be explored.

Another approach that has been put forward to explain the poorer figurative language competence in ASD is linked to general cognitive mechanisms that characterize the autistic phenotype. It has been claimed that increased semantic integration demands, as well as a tendency to weak central coherence, may underlie the difficulties observed in figurative language competence in ASD (Vulchanova et al., 2015). Vulchanova et al. (2015) point to various top-down abilities that may explain the difficulties in figurative language comprehension in ASD. These abilities are associated with the inability to use information adequately, including evaluating the plausibility of events, assessing what is relevant, and combining information arising from different modalities.

Another argument explaining the poorer metaphorical comprehension in ASD is related to the task properties. A recent meta-analysis (Kalandadze et al., 2019) noted that individuals with ASD may demonstrate more difficulties in tasks requiring integration of multiple modalities (although this observation was based on the results of only two studies). In particular, while tasks requiring verbal explanations appeared to be the most demanding task for participants with ASD, decisions about meaningfulness appeared to be the easiest. Given the importance of response format in metaphor comprehension, this might be relevant for studies comparing individuals with ASD to individuals with TD on tasks requiring metaphor generation.

Figurative language deficits in ASD are linked to pragmatic abilities (Happé, 1991, 1993), that include the ability to distinct between what is said and the intended meaning (Grice, 1975). Recent literature point to a larger debate on whether or not there is a pragmatic deficit in ASD (Deliens et al., 2018; Mazzaggio and Surian, 2018) with several scholars suggesting that the extent of pragmatic difficulties in ASD seems to vary depending on the specific kind of the required inference and on the possible mechanisms involved (Kissine, 2016; Andrés-Roqueta and Katsos, 2017). A recent study tested the unique pragmatic profile of individuals with ASD (Schaeken et al., 2018) using both a binary task (accept or reject a statement) and a novel ternary task (with a middle answer option). The study examined informativeness and scalar implicatures (linguistic expressions such as: all; many; some; must; should; may) in children with ASD. The results of the ternary task revealed that children with ASD tended to either fully agree or fully disagree with under informative statements, unlike their TD peers who demonstrated preference for intermediate options. These findings indicate deficient scalar processing in ASD, thus contributing to the understanding of the pragmatic profile of ASD and the importance of response format.

Consistent with the heterogeneous nature of figurative language, recent studies indicate that individuals with ASD do not necessarily differ from their TD peers in all aspects of figurative language processing. Specifically, metaphors are more difficult to comprehend for individuals with ASD, compared with their TD peers, than were irony and sarcasm (Kalandadze et al., 2018). On the other hand, Kasirer and Mashal (2014) demonstrated that the ability to identify novel semantic connections between apparently unrelated concepts (an ability observed when assessing novel metaphor comprehension) is not necessarily impaired in adults with ASD (Mashal and Kasirer, 2011; Hermann et al., 2013; Kasirer and Mashal, 2014, 2016; Melogno et al., 2017). Additional support for this finding was obtained from a study that examined adults with Asperger’s syndrome engaging in a semantic judgment task (Hermann et al., 2013). The study, which had participants make rapid decisions about the literal meaning of given sentences, found that the processing of novel metaphors among the Asperger’s group was similar to the performance of their age-matched peers with TD. Thus, there is evidence that comprehension of non-lexicalized figurative expressions is not impaired among adults with ASD.

Similarly, the ability of children with ASD to comprehend non-lexicalized figurative language appears consistent with those of adults with ASD (Kasirer and Mashal, 2014). Zheng et al. (2015) showed that comprehension of novel metaphors and metonyms among Chinese children with ASD did not differ from the performance of the comparison TD group (see also Melogno et al., 2019). Moreover, a study by Kasirer and Mashal (2016) of children with ASD demonstrated that while their comprehension of conventional metaphors was lower than their TD peers, no group differences were observed in novel metaphor comprehension; furthermore, while the ASD group *generated* less conventional metaphors (e.g., feeling lonely is…an abandoned dog), they also generated more creative and novel metaphors (e.g., feeling successful is…taking an elevator to heaven) (Kasirer and Mashal, 2016). These findings suggest a potentially unique capability for verbal creativity in ASD. Since metaphor generation is considered the most powerful source of linguistic innovation, it provides a fruitful context for studying creativity (Levorato and Cacciari, 2002; Silvia and Beaty, 2012).

Creativity can be defined as the ability to generate new, adaptive ideas or novel solutions to problems that may have substantial value to humanity (Runco and Jaeger, 2012). Creative thinking relies on divergent thinking (Milgram and Livne, 2006; Runco, 2010). Divergent thinking is the ability to produce multiple ideas and associations to a given problem. This ability consists of fluency (number of responses), flexibility (shifting between different ideas), originality (uniqueness of ideas), and elaboration (amount of detail). The ability is assessed by open-ended tests that establish the fluency while generating as many multi solutions as possible (Guilford, 1959).

Imagination plays an important role in a creative mind. Some studies report that individuals with ASD tend to fail in imaginative tasks (Jarrold et al., 1993; Craig and Baron-Cohen, 1999; Burton, 2010). However, Liu et al. (2011) had demonstrated that successful performance on tasks that involve creativity and originality does not necessarily involve imagination. In their study, they asked children with and without Asperger’s syndrome to complete incomplete figures in an original way, as well as to provide the figures with a title. The completed drawings were assessed on domains of fluency, openness, flexibility, originality, and elaboration. The results showed that the children with Asperger’s syndrome demonstrated greater elaboration and originality than their age-matched peers with TD. A recent study (Hetzroni et al., 2019) also investigated creative thinking abilities among children aged 9–11 with ASD and TD. The study compared performance on two different creativity tests: a pictorial divergent creativity test (Pictorial Multiple Solutions, PMS) and a mathematical convergent creativity test (Creating Equal Number, CEN). Performance on both tasks was scored on fluency, flexibility, originality, and creativity. The results indicated that the children with ASD performed similarly to the TD group on both creativity tests. Additionally, while the children with TD demonstrated better performance on fluency and originality on the PMS test, children with ASD slightly outperformed the TD group on the mathematical CEN test. Thus, this body of research may suggest that individuals with ASD possess specific creative capabilities.

Creativity is usually tested by verbal or figural output (Goff and Torrance, 2002). Figural creativity may be assessed by the Karmiloff-Smith’s nonexistent object drawing task (Karmiloff-Smith, 1990). The nonexistent object drawing task examines imagination through visual drawing. Children are asked to draw an object that does not exist, alternately described in different ways to them: an object they invent, an object they have never seen before, a strange object, or an object with something funny or odd about it (Karmiloff-Smith, 1990; Spensley and Taylor, 1999; Adi-Japha et al., 2010). Karmiloff-Smith (1990) showed that when children were asked to draw an “impossible man” who does not exist (similarly with an animal and a house), 4- to 6-year-olds tended to make size, shape, or deletion changes to elements within their drawings, whereas 8- to 10-year-olds exchanged the position of elements, added extra elements from the same category (i.e., same-category insertion, such as a man with four legs), or included cross-category insertions that combine two different elements (e.g., a house with tails). The former three categories (changes of size, shape, deletion) were classified as representing simpler changes, while the latter three categories (element exchange, same-category insertion, cross-category insertion) were classified as representing more complex changes.

Previous studies demonstrated poorer performance among participants with ASD on Karmiloff-Smith’s (1990) “draw an impossible man” task (Scott and Baron-Cohen, 1996; Craig et al., 2001; Low et al., 2009). For instance, Low et al. (2009) found that children with ASD scored lower, compared to children with TD, on the drawing task. Only 59% of children in the ASD group succeeded in generating a picture containing imaginative features, compared to the 93% of children in the TD group who succeeded in doing so. Generativity and planning abilities were, in particular, lower among the ASD group.

However, a recent study that examined children (5–14 years old) using the Karmiloff-Smith’s (1990) drawing task and tests of other cognitive, verbal, and executive abilities, found that children with ASD produced drawings of houses with an equal proportion of imaginative features as the control group (Ten Eycke and Müller, 2018). However, the ASD group generated drawings of *people* with significantly lower proportions of imaginative features. The results also point to a different cognitive strategy in imaginative drawing used by the children with ASD compared to their peers with TD. In the TD group, executive functioning and cognitive-perceptual processing styles (as assessed by the Embedded Figure Task and optical illusions task) predicted imaginative drawing, but these associations were moderated by mental age. For children with ASD, only executive functioning significantly predicted imaginative drawing, which may imply that these group differences are explained by developmental delays associated with ASD. Thus, children with ASD may employ a unique cognitive strategy such as “think in pictures” (i.e., use visual rather than verbal representations) in imaginative drawing.

Creativity is also assessed by verbal output (Goff and Torrance, 2002). Producing novel metaphors includes the ability to “think outside the box,” break common thinking patterns, use original rules of thinking and think abstractly (Dietrich, 2004). Evidence suggests that generation of novel metaphors among adults with TD relies on higher cognitive resources, such as fluid intelligence and executive processes (Beaty and Silvia, 2013). It has been shown that fluency of ideas (as assessed by the “alternate uses” task) contributes to creative performance beyond similarities, cognitive abilities, executive functions, verbal abilities, and age in children with TD (Kasirer and Mashal, 2018). Alternatively, it has also been reported that phonemic fluency contributes to creative performance (as assessed by novel metaphor generation) in children with ASD (Kasirer and Mashal, 2016). Phonemic fluency involves strategic searching, retrieval abilities, response initiation monitoring, shifting, and flexibility (e.g., Kavé et al., 2010). The intact creative abilities among individuals with ASD in certain types of creative tasks apparently rely on different abilities than individuals with TD.

It is well-established that without creativity there are difficulties adapting to a world that is changing at an unprecedented pace (Akltas, 2017). Evidence suggests that there is a significant relationship between creativity and emotional, psychological, and social well-being (Tamannaeifar and Motaghedifard, 2014). Therefore, it is important to shed light on creative abilities in ASD. Consensus from studies that tested creativity in ASD has heretofore been inconclusive. Whereas previous studies have shown low creative abilities in certain tasks (Scott and Baron-Cohen, 1996; Craig et al., 2001; Low et al., 2009), recent studies demonstrate similar creative abilities (Hetzroni et al., 2019) or greater verbally creative abilities in ASD as compared to individuals with TD (Kasirer and Mashal, 2014, 2016). The current study compares for the first time two generative creative ability tasks requiring different capabilities: a verbal and a non-verbal imaginative drawing task. The latter is not based on verbal and social abilities that are core deficits in autism. These tasks may serve as valuable tools in evaluating creative ability in ASD and the cognitive mechanisms underlying these abilities in TD. Thus, the goal of utilizing both creativity tasks in our research was to help to explore the capabilities of this population that may have been inadvertently eclipsed by previous research. Finding similar or greater creative ability among those with ASD compared to those with TD would demonstrate that those with ASD are indeed able to generate novel ideas, a necessary skill for adapting to a dynamically changing world.

The overarching goal of the current study was, therefore, to examine verbal and non-verbal (figural) creativity in children with ASD compared to children with TD. In particular, its goal was to examine which type of changes each group would make on the “draw a non-existent object” task (changes in element shape or size, whole shape changes, deletion of elements, same-category insertion, orientation/position change and cross-category insertion). In addition, we examined the differential contribution of language abilities (novel or conventional metaphor comprehension, vocabulary), as well as cognitive and executive functioning, on creative performance in each study group. Based on previous studies (Kasirer and Mashal, 2014, 2016, 2018), we expected that participants with ASD would be able to generate more novel metaphors than their TD peers. In addition, based the unique style of thinking that characterizes people with ASD (Happé, 1999) and their tendency to think less conventionally we expected that children with ASD would generate more novel metaphors. Furthermore, mind-blindness makes them ignoring the addressee, focusing on their own thoughts (Happé and Vital, 2009), and may thus lead to the generation of expressions that are less conventional (Liu et al., 2011). We also hypothesized that children with ASD would exhibit similar abilities in imaginative drawing to their peers with TD (Ten Eycke and Müller, 2018). Finally, we hypothesized that higher executive functioning and, in particular, higher phonetic fluency scores, would contribute to greater metaphor generation in both the ASD (Kasirer and Mashal, 2016) and the TD group (Kasirer and Mashal, 2018) with additional reliance on non-verbal intelligence among children with TD (Beaty and Silvia, 2013).

## Materials and Methods

### Participants

Seventy-nine children aged 11–15 years old were recruited for the study and included 40 children with ASD (35 boys and 5 girls) ranging in age from 11 to 14 years old, and 39 children with TD (28 boys and 11 girls) ranging in age from 11 to 15 years old. The groups did not differ in age [*t*(77) = 0.11, *p* = 0.91] or in gender [χ^2^(1) = 3.01, *p* = 0.08]. All children were native Hebrew speakers. The children with TD were recruited from elementary schools and junior high schools. The participants with ASD were recruited from integrated classes within elementary schools and junior high schools. Diagnosis was made by a community psychiatrist, in line with the DSM-IV-TR criteria (American Psychiatric Association [APA], 2000). Participants also completed the Social Communication Questionnaire (Berument et al., 1999). The questionnaire includes three domains of functioning: reciprocal social interaction, language and communication, and repetitive and stereotyped behaviors. All participants with ASD scored above 15 on this questionnaire, thus verifying the clinical diagnoses.

All participants scored within the age-appropriate range on the screening tests (see Table 1). All parents received a letter describing the aims of the study and provided signed informed consent before the beginning of the study. The study was explained to all of the children in a simple fashion and they were asked if they wished to participate; all of the children assented to participate. The study was approved by the Israeli Ministry of Education. Participant recruitment was conducted in accordance with institutional research guidelines.

**TABLE 1:** Mean age and screening test scores per group.

	TD (*n* = 39)		ASD (*n* = 40)				
		
	*M*	*SD*	*M*	*SD*	Welch *t*	*p*	*d*
Age	11.76	1.88	11.62	2.31	0.11	0.907	0.025
TONI-3	31.12	6.96	32.15	6.30	0.68	0.496	0.155
WISC Vocabulary	46.33	5.69	44.62	6.18	1.27	0.206	0.299
Picture naming	43.97	2.81	43.72	3.29	0.36	0.719	0.082

### Materials and Design

#### Screening Tests

Several screening tests were used to assess verbal and non-verbal abilities (see Table 1).

*Test of Non-verbal Intelligence − Third Edition* (TONI-3; Brown et al., 1997). The TONI-3 consists of 45 black-and-white items arranged according to degree of problem solving difficulty. This test assesses the subject’s ability to solve abstract/figural problems without depending on the verbal skills of the subject.

*Wechsler Intelligence Scale for Children* (WISC-IV^HEB^; Wechsler, 2003) – *Vocabulary*. The vocabulary subtest from the Wechsler Intelligence Scale for Children was utilized to assess each participant’s level of vocabulary.

*Picture-Naming Test* (Kavé, 2005) − *Hebrew*. This test consists of 48 black-and-white line drawings; the participant is instructed to name, in one word, the object in the picture. This test is designed to assess naming abilities.

As can be seen in Table 1, the groups did not differ by age, or performance on the TONI-3, WISC vocabulary, or picture naming test.

#### Executive Function

Executive function (EF) was assessed by the Ambiguous Word Meaning Generation Test (AMGT; Mashal and Kasirer, 2011) and the phonemic and semantic fluency tests (Kavé, 2005).

*Ambiguous Word Meaning Generation Test* (AMGT). This test examines the ability to activate different meanings of ambiguous words (e.g., bank) and to shift between them. Participants are presented with a list of 20 short unbiased sentences that end with ambiguous words (e.g., Look at this bank). Participants are asked to say aloud all meanings of the final word. The score is the number of correct responses provided for all ambiguous words.

##### Phonemic fluency

This test investigates the ability to flexibly search among different words in a given amount of time. According to Kavé (2005), phonemic fluency is assessed by obtaining the number of words produced in one minute for the letters bet (b), gimmel (g), and shin (sh). We used a sum score of the words generated for all three letters.

##### Semantic fluency

This test investigates the ability to flexibly search among different categories in a given amount of time. According to Kavé (2005), semantic fluency is assessed by summing the number of words generated in one minute for three semantic categories: animals, fruits and vegetables, and vehicles. We used a sum score of the words generated in all three categories.

#### Metaphor Comprehension and Generation

*Novel and Conventional Metaphor Comprehension* (Mashal and Kasirer, 2011). This questionnaire, developed by Mashal and Kasirer (2011), tested comprehension of two-word conventional metaphors (e.g., *thunderous silence*) and novel metaphors (e.g., *pure hand*). For each metaphoric expression, four alternative interpretations are offered: a correct metaphoric interpretation, a literal interpretation, an unrelated interpretation, and the phrase “this expression is meaningless.” Participants are instructed to choose the best answer. The questionnaire consists of 20 items and scores are the sum of all correct answers. Examples of two conventional metaphors used in the questionnaire: *Heart of the matter*: (1) This phrase has no meaning; (2) A love story; (3) An important organ in the human body; (4) The essence of things (correct response). *Thundering silence*: (1) Being silent; (2) The quiet before the thunder; (3) Lack of response that expresses dissatisfaction (correct response); (4) This phrase has no meaning. Examples of two novel metaphors: *Manufactured smile*: (1) An unreal, fake smile (correct response); (2) Someone who smiles while cheating on someone else; (3) Artificial food; (4) This phrase has no meaning. *Pure hand*: (1) Long limb; (2) White fingers; (3) A person who does no evil deeds (correct response); (4) This phrase has no meaning.

This test provides two measures: novel metaphor comprehension and conventional metaphor comprehension. The range of score for novel metaphor comprehension was 0–10, and for conventional metaphor comprehension was 0–10.

#### Figural and Verbal Creativity

##### Creative metaphor generation

This test examines verbal creativity. The test presents 10 “concepts” that were used in a recent study (Kasirer and Mashal, 2016) based on a previous creative metaphor generation study of Levorato and Cacciari (2002). Five of the concepts are presented to the participants as a metaphor (e.g., love is____) and 5 are presented as a simile (e.g., feeling happy is like____). The participants are asked to generate a new way of expressing the meaning of the concept. They are also encouraged to create a new expression, rather than simply rephrasing the one with which they are presented. Two judges code the data independently, determining whether each expression is literal or figurative; when there is a disagreement, a third judge is asked to make a determination. A metaphor is considered creative if it is an unfamiliar, unique, and novel (non-literal) expression (e.g., “*Feeling worthless is*…*a totally smashed lemon”*); a conventional metaphor is a familiar expression or idiom (e.g., “*Feeling embarrassed is* …*being caught with my pants down”*), and a literal response is rephrasing the concept or using a simple non-figurative description (e.g., “*Feeling successful is*…*a winning”*). The responses are classified into: (1) creative responses; (2) conventional responses; (3) literal responses; or (4) unrelated or inappropriate responses. The judges were blind to the study hypotheses and group affiliation for each response. The general intra-class correlation (ICC) was high, *ICC* = 0.97 (participants with ASD: *ICC* = 0.98; participants with TD: *ICC* = 0.96). Verbal creativity was calculated as the total number of creative, appropriate responses. The range of score for novel metaphor generation was 0–10.

##### Non-existent object drawing test

This test examines non-verbal (figural) creativity (Karmiloff-Smith, 1990). The children are first asked to draw a house. After completing their drawing, they are requested to draw ‘a house that does not exist’. Several phrasings are used to enable the children to understand the task: “a house you invent,” “a house you have never seen before,” “a strange house,” “a house with something funny/odd,” and “a make-believe/pretend house.” After drawing the non-existent house, the children are asked to verbalize why such a house does not exist. Following the procedure developed by Karmiloff-Smith (1990), two independent judges score the categories of changes according to (a) no change, (b) change in element shape or size, (c) whole shape changes, (d) deletion of elements, (e) insertion of new (same-category) elements (e.g., a house with two chimneys), (f) position or orientation changes, and (g) cross-category insertions (e.g., a house with a tail). Categories are judged as binary variables (appear/do not appear) for each drawing. Note that categories (b) – (d) are considered “simple changes” while categories (e) – (g) are considered “complex” as they involve higher executive functions. Each category is assigned values of 0 (does not appear at all) – 2 (appears in both non-existent drawings). The simple/complex categories are assigned values of 0 (does not appear at all) – 6 (all three categories appear in both drawings). Each drawing was classified as showing change if the non-existent house violated “house-ness”. A second house similar to the first, a bigger and smaller house, or a house and a different, yet conventional, house that did not include non-existent features, were classified as not showing change, even if the verbal response indicated some difference (e.g., “this house does not exist because it’s shaped like ice cream in make-believe”). Drawings were rated by two independent rates. Raters were trained to score the test based on sample drawings from the present and previous studies (Adi-Japha et al., 2010). Cohen’s *k* coefficients across all drawings for inter-rater agreement were above 0.8 (*p* < 0.001) in all change categories. Where necessary, disagreements were settled by discussion. The same process was followed for an animal drawing.

### Analyses

First, we compared the two groups on creative metaphor generation and on non-existent object drawing performance. Due to violation of normality assumptions, non-parametric statistics were used, and groups were compared using the Mann-Whitney test. We then calculated Spearman correlations between scores on the Creative Metaphor Generation task and the non-existent object drawing task (namely, the sum of scores representing use of (a) “simple” changes, (b) “complex” changes, and (c) cross-category insertion changes that the children made across the two drawings). Next, before performing the regression analysis on the creative metaphor generation scores, Spearman correlations were calculated between the scores on the Creative Metaphor Generation test and scores on the cognitive tests (TONI-3, picture naming test, vocabulary, AMGT, fluency tests, novel metaphor comprehension and conventional metaphor comprehension). The same was done between the cross-category insertion scale and cognitive tests. Finally, based on the correlation results, a hierarchical linear regression analysis was performed in each group separately. The bootstrap procedure was used in the regression analyses to account for violations of normality assumptions. The resampling procedure creates many (we used 1000) simulated samples of the original sample’s size, each with its own properties, such as the mean. The sampling distribution was based on the bootstrapping procedures that use the distribution of the sample statistics across the simulated samples. The simulated distribution converges well to the true distribution function because the sample size is large. It then follows that the bootstrap variance is a good estimate of the true variance of the population mean (Efron and Tibshirani, 1993).

## Results

The results of the executive function tests (phonemic and semantic fluency), the AMGT, the metaphor comprehension test (conventional and novel), and the creativity test (novel metaphor generation) are presented in Table 2.

**TABLE 2:** Descriptive statistics: Verbal measures.

	TD (*n* = 39)		ASD (*n* = 40)				
		
	*M*	*SD*	*M*	*SD*	*Z*	*p*	d
Phonemic fluency	27.84	7.20	22.47	9.19	2.69	0.007	0.656
Semantic fluency	42.92	8.26	38.52	8.86	2.02	0.045	0.519
AMGT	14.15	6.45	10.35	6.46	3.03	0.002	0.596
Novel metaphor	7.56	1.88	7.07	2.39	0.72	0.471	0.230
Conventional metaphor	7.89	2.11	6.17	2.23	3.63	0.000	0.802
Verbal creativity	1.94	1.58	3.07	2.06	2.49	0.014	0.624

*AMGT = Ambiguous Word Meaning Generation Test; Novel metaphor = novel metaphor comprehension; Conventional metaphor = conventional metaphor comprehension; Verbal creativity = Creative metaphor generation.*

As can be seen in Table 2, children with TD scored higher on conventional metaphor comprehension, but not on novel metaphor comprehension, than their age and language-matched ASD peers. In addition, children with TD scored higher than their peers with ASD on the AGMT and the other executive function tests (indicated by the fluency scores). However, as hypothesized, children with ASD generated more creative metaphors than their peers with TD.

### The Non-existent Object Drawing Task

Because all children were older than 8 years of age, we did not expect group differences in cognitive flexibility to emerge in the simpler change categories of the non-existent object drawing task (see Table 3). Nevertheless, in light of the findings of Adi-Japha et al. (2010), we expected that differences would be reflected in the cross-category insertion scale which is relatively easier to produce among the more complex change categories of the task (such as same-category insertion and orientation/position change). This was, therefore, the main variable of interest.

**TABLE 3:** Non-existent object drawing task.

	TD (*n* = 39)		ASD (*n* = 40)				
		
	*M*	*SD*	*M*	*SD*	*Z*	*p*	*d*
**Simple Change Categories**							
Deletion	0.31	0.46	0.55	0.71	1.40	0.160	0.405
Element’s Shape	0.74	0.81	0.60	0.74	0.75	0.451	0.185
Whole Shape	0.87	0.76	0.97	0.83	0.54	0.584	0.131
Sum of Simple Change Categories	1.87	1.26	2.07	1.38	0.72	0.470	0.155
**Complex Change Categories**							
Orientation/Position	0.25	0.49	0.20	0.40	0.38	0.700	0.126
Same-Category Insertion	0.46	0.55	0.30	0.46	1.31	0.190	0.320
Cross-Category Insertion	0.71	0.72	1.12	0.82	2.23	0.025	0.532
Sum of Complex Change Categories	1.43	0.93	1.62	1.00	0.90	0.369	0.197
Sum of All Change Categories	3.30	1.41	3.70	1.60	1.17	0.242	0.262

**FIGURE 1:**
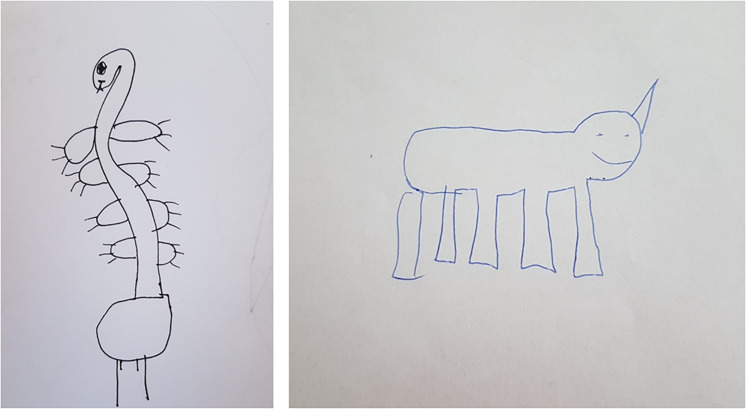
The non-existent object drawing task. Left panel: Example of cross-category insertion by 11 year old child with ASD (a giraffe with a snake). Right panel: Example of same-category insertion by 11 year old child with TD (a dog with five legs).

As can be seen in the Table 3, the ASD group used more cross-category insertions (e.g., a house with a tail) then their peers with TD. See Figure 1 for cross- and same-category insertion examples. See also the Appendix for more examples of verbal and figural responses provided by ASD children. To facilitate comparison with previous studies, the other change categories are specified as well (see Table 3). No significant differences between the groups were observed in the other categories.

### Correlation Analyses

Performance on generation of creative metaphors correlated with the overall sum of category changes on the non-existent object drawing task (i.e., children who generated more creative metaphors used more change categories in their drawings, *rs*(39) = 0.34, *p* = 0.033, *rs*(40) = 0.60, *p* < 0.001, for children with TD and ASD, respectively). Furthermore, generation of creative metaphors correlated with use of simple change categories [*rs*(39) = 0.41, *p* = 0.011, *rs*(40) = 0.53, *p* < 0.001, for children with TD and ASD, respectively]. However, no correlation emerged between generation of creative metaphors and use of cross-category insertions on the drawing task.

### Correlations With Cognitive and Verbal Scores

Next, we correlated creative metaphor generation, as well as cross-category insertion, with the TONI-3, picture naming test, vocabulary subset of the Wechsler scale, AMGT score, fluency tests, novel metaphor comprehension, and conventional metaphor comprehension scores, using *p* < 0.01 as a cutoff due to multiple correlations.

For the generation of creative metaphors, significant associations with cognitive and verbal measures emerged for the TD group with the TONI-3, *rs* = 0.53 (*p* = 0.001), and the vocabulary scores, *rs* = 46 (*p* = 0.003). In the ASD group, significant associations emerged for phonetic fluency *rs* = 0.48 (*p* = 0.002), novel metaphor comprehension, *rs* = 0.48 (*p* = 0.002) and conventional metaphor comprehension, *rs* = 0.45 (*p* = 0.004). No significant associations emerged for the cross-category insertion scores with any variable.

### Regression Analysis

Next, due to the small sample size and following the correlation analyses, the generated creative metaphor scores in the TD and ASD groups were subjected to a hierarchal bootstrapped regression analysis with age and gender in the first step, and variables that significantly correlated with the generation of creative metaphors in the following steps. Due to a high level of correlation between the TONI-3 and the vocabulary scores for the TD group [*rs*(39) = 0.48], a stepwise procedure was used and the TONI-3 scores were entered in the second step, followed by the vocabulary scores. Only the TONI-3 scores contributed to the model (see Table 4). For the ASD group, novel metaphor comprehension and conventional metaphor comprehension were highly correlated [*rs*(39) = 0.65]. Therefore, phonetic fluency and novel metaphor comprehension were entered in the second step, followed by the conventional metaphor comprehension. However, the latter variable did not significantly add to the model; therefore, only the phonetic fluency and novel metaphor comprehension remained (see Table 4).

**TABLE 4:** Regression analyses explaining the variance in the generation of creative metaphors.

	TD			
	
	β	*SE*	Bias (β)	*p*
**Step 1**				
Age	0.37	0.12	–0.003	0.007
Gender	0.03	0.53	–0.003	0.950
ΔR^2^	0.21			
**Step 2**				
Age	0.25	0.13	0.013	0.072
Gender	0.22	0.51	0.012	0.667
TONI	0.079	0.040	–0.004	0.054
ΔR^2^	0.11			
Total R^2^	0.32			

	**ASD**			
	
	**β**	***SE***	**Bias (β)**	***p***

**Step 1**				
Age	0.34	0.12	–0.007	0.011
Gender	–0.99	0.52	0.002	0.056
ΔR^2^	0.17			
**Step 2**				
Age	0.17	0.12	0.004	0.132
Gender	–0.64	0.71	0.000	0.357
Phonetic Fluency	0.11***	0.03	0.003	0.001
Novel metaphor comprehension	0.27*	0.11	0.001	0.018
ΔR^2^	0.25			
Total R^2^	0.42			

*p < 0.05; ***p < 0.01.

Because no associations emerged between the cross-category insertion scores and other cognitive and verbal measures, we did not conduct a regression analysis for this variable.

## Discussion and Implications

The current study investigated verbal and figural creativity among children with ASD compared to their age and language-matched TD peers. One major finding that emerged from the current study is that children with ASD outperformed children with TD on verbal creativity (as assessed by novel metaphor generation). Second, in the figural creativity task, children with ASD used more cross-category insertions (e.g., a house with a tail) than their age and language-matched peers with TD. Finally, although the metaphor generation and non-existent drawing tests both examine creative thinking, this study further assessed the different skills required for each type of creativity within each group of subjects.

Participants with ASD exhibited better performance on novel, original metaphor generation (e.g., *Feeling worthless is*…*evaporated water*) than their age and language-matched TD peers, who mostly generated conventional metaphors (e.g., *Feeling angry is*…*a volcano*).

These findings corroborate previous study findings suggesting a unique verbal ability of generating novel, original metaphors among those with ASD (Kasirer and Mashal, 2014, 2016). It has been suggested that children with ASD exhibit unique verbal associations probably reflecting their non-conventional style of thinking that is not limited by lexicalized knowledge as compare to children with TD (Kasirer and Mashal, 2016). A qualitative recent study (Morra, 2016) examined how individuals with ASD (aged 14 to 60 years) perceive their capacity to process metaphor. ASD quotes were collected from online posts in planned network communication for people with ASD and others neurological disorders (conducted from 2007–2016). The results of the study were inconclusive, showing that while some ASD writers provided examples of creative metaphors that were understood by many of their recipients, others claimed that TD individuals seemed not to understand the metaphors they coined. Morra (2016) suggested that people with ASD generate metaphors relying on their experience and special interests, similar to TD individuals, but because of their unusual area of interest and unique experience, they may create novel metaphors that are not appropriate or rare leading to misunderstanding. It should be noted that in the current study no inappropriate or misunderstood answers were observed.

The current study tested both metaphor comprehension and metaphor generation. Similar to metaphor comprehension, metaphor generation also requires searching for semantic connections between apparently unrelated concepts. Despite this similarity, studies that tested metaphor generation are remarkably scarce compared to studies of metaphor comprehension. Indeed, many researchers of psycholinguistics (Lakoff and Johnson, 1980; Gibbs, 1994; Kintsch, 2000) and neuroscience (Rapp and Goldrick, 2004; Mashal et al., 2007; Bambini et al., 2011) have studies the underlying cognitive processes and neural substrates of metaphor comprehension; Nevertheless, very little is known about metaphor generation. Recently, several scholars have begun to pinpoint the cognitive abilities underlying metaphor generation (Chiappe and Chiappe, 2007; Beaty and Silvia, 2013; Kasirer and Mashal, 2018). Chiappe and Chiappe (2007) found an important role of verbal knowledge and working memory functions in both metaphor comprehension and generation. In a further examination, Kasirer and Mashal (2018) used regression analyses to compare the differential contribution of language (naming and vocabulary), executive functions (AMGT, fluency, and mental flexibility), and cognitive abilities (non-verbal intelligence test) on novel metaphor comprehension and metaphor generation. The results indicated that the comprehension of novel metaphors was best predicted by mental flexibility. The generation of novel metaphors, on the other hand, was best predicted by non-verbal intelligence. However, the results were obtained for participants with TD *and* ASD, but not for each group separately.

Our results also show that novel metaphor generation is linked to novel metaphor understanding in the ASD, but not in the TD group, suggesting that metaphor competence might be sovramodal in ASD. Although this idea is novel and was not tested before, in a previous study (Mashal and Kasirer, 2012) a principal component analysis (PCA) was performed to examine the classification of several tests that include, novel metaphor comprehension, conventional metaphor comprehension, idiom comprehension, visual metaphor comprehension, synonyms, similarities, fluency tests, AMGT, and reading test in a group of children with ASD and TD. The results showed that children with ASD demonstrated a different pattern of clusters. Whereas the first component of the TD group included the synonyms, the AMGT, and the reading test, the first component of the ASD group included the conventional metaphor, the novel metaphor comprehension test, and the idioms. Visual metaphor comprehension was clustered with novel metaphor comprehension in the last component of the TD group. Thus, although the study (Mashal and Kasirer, 2012) did not use a metaphor generation test, the results show that figurative language tests (conventional metaphors, novel metaphors, idioms) cluster together in ASD. Given the paucity of studies that tested both metaphor comprehension and generation future study should test whether metaphoric competence (regardless the tasks and including generation tasks) constitutes a sovramodal ability in ASD.

Regarding the neural substrates associated with metaphor comprehension and generation, it has been shown that metaphor generation was associated with increased activation in several left-hemisphere brain areas, including the left angular gyrus (AG), the left dorsomedial prefrontal cortex (DMPFC), and the posterior cingulate cortex (PCC), areas that were implicated in both comprehension and generation of metaphors (Benedek et al., 2014). These shared activations probably reflect the common mechanism required for both metaphor comprehension and generation as both processes require the activation of semantic information needed to integrate distantly related concepts. Thus, it seems that metaphor comprehension and generation share the same left hemisphere brain regions but they differ in their underlying cognitive mechanisms. Given the remarkably scarce number of studies that compared metaphor generation and comprehension, future studies are necessary to address this topic.

As expected, and in line with previous studies showing difficulties with metaphoric language comprehension in those with ASD (e.g., Happé, 1993; MacKay and Shaw, 2004; Rundblad and Annaz, 2010; Mashal and Kasirer, 2011; Melogno et al., 2017; Kalandadze et al., 2018), children with ASD in the current study scored lower on conventional metaphor comprehension than children with TD. However, no group difference was observed in novel metaphor comprehension, attesting to the intact ability among those with ASD to make novel semantic connections. Unlike conventional metaphors that are coded in the mental lexicon, novel metaphor interpretation is not coded and, therefore, not dependent on previous knowledge. According to Melogno et al. (2012) children with ASD appears to have intact understanding of novel metaphors possibly because they rely on their uncommon phonological or semantic associative skills rather than relying on lexicalized verbal knowledge. The ability to understand novel but not conventional metaphors in ASD is also consistent with previous findings of metaphor comprehension in ASD (Mashal and Kasirer, 2011; Hermann et al., 2013; Kasirer and Mashal, 2014, 2016; Melogno et al., 2017). In the current study, a multiple-choice questionnaire was used to assess metaphor comprehension. Using such methodology could affect the results if participants are either distracted by alternative answers, or find the task easier than previously utilized tasks that require sentence completion (Norbury, 2005), ask comprehension questions about metaphoric stories (Rundblad and Annaz, 2010), or involve performing a semantic judgment task (Hermann et al., 2013). It has been suggested that metaphor comprehension in general is not unequivocal deficient in ASD with some people with ASD showing difficulty in metaphorical comprehension and a tendency toward literal interpretation, while others have intact metaphorical language competence (e.g., Morra, 2016).

Another finding of the current study is that, contrary to our hypothesis, we found differences in the figural creativity task between the study groups, with participants with ASD using more cross-category insertions (e.g., a house with a tail). As the non-existent object drawing task measures the kinds of changes the participant makes to a drawing of an animal or house that does not exist in reality, it relies on executive functions (Spensley and Taylor, 1999) such as planning and flexibility (Adi-Japha et al., 2010), as well as imaginative ability (Low et al., 2009). Earlier studies have reported decreased imagination in ASD (Lewis and Boucher, 1991; Craig and Baron-Cohen, 1999; Hetzroni et al., 2019), attributing this deficit to poorer cognitive flexibility (Craig and Baron-Cohen, 1999). However, in the current study, the greater success of children with ASD to make cross-category insertions, which involves linking components of different categories of objects, appears to suggest intact flexibility, at least in terms of simple modifications to drawings (Freeman and Adi-Japha, 2008).

Furthermore, the ASD cohort in our study did not differ from their TD counterparts on the performance of other age-appropriate types of changes on the non-existent object drawing task (e.g., position or orientation changes, insertion of a same category element); such modifications require early planning and inhibition. While Robinson et al. (2009) found group differences in inhibition, planning, and self-monitoring, no differences in cognitive flexibility were found between the groups. It seems that despite potentially compromised planning capabilities in ASD (Hill, 2004; Robinson et al., 2009), flexibility is preserved for this particular (drawing) task. Thus, while the evidence for executive dysfunction in ASD remains equivocal, their uncommon and associative style of thinking (Happé, 1993) may, in fact, contribute to their success in performing creative tasks. Indeed, the imaginative drawing process involves the manipulation of representations to produce something original, similar to the metaphor generation task that elicits generation of novel concepts. This may signify enhanced capabilities in these areas among those with ASD.

Of interest, however, the correlation analyses indicated that although the metaphor generation task correlated with the overall use of change categories (and, in particular, with the use of simple change categories), no correlation was found between the metaphor generation task and use of cross-category changes. This finding suggests that the generation of creative metaphors and the use of cross-category insertions rely on different abilities. The use of cross-category insertion, which is an age-related complex change-category, requires flexibility (but to a lesser extent planning and inhibition; Adi-Japha et al., 2010). Metaphor generation, conversely, relies on cognitive processes such as fluid intelligence as well as executive processes that include planning and inhibition (Nusbaum and Silvia, 2011; Beaty and Silvia, 2013; Kasirer and Mashal, 2016). The findings emerging from the regression analyses indeed pinpoint a different pattern of associations between executive function, metaphor comprehension, verbal and cognitive functions abilities, and performance on the two creativity tasks. Whereas fluid intelligence (assessed by the TONI-3; Brown et al., 1997) contributed marginally to novel metaphor generation in the TD group, a finding supported by previous research (Silvia and Beaty, 2012), none of these tests contributed to the explained variance on the drawing test.

Finally, our results show that the ASD and TD groups differ in the cognitive abilities they used to perform the metaphor generation task. Whereas phonemic fluency, which is a language-based executive function test, contributed to novel metaphor generation in the ASD group (in addition to novel metaphor comprehension), fluid intelligence contributed to the novel metaphor generation task in the TD group. A recent study (Kasirer and Mashal, 2016) also reported the contribution of phonemic fluency, beyond age and gender, to the generation of creative metaphors among children and adolescents with ASD. Phonemic fluency demonstrates strategic searching, response initiation, monitoring, shifting, and flexibility (e.g., Kavé et al., 2010). Likewise, generating novel creative metaphors shares the ability to use uncommon and perhaps surprising patterns of thinking (Dietrich, 2004).

The link between executive functioning and metaphor comprehension deserves special attention. To the best of our knowledge, there are few previous studies relating executive functioning to metaphor comprehension. Several studies have linked metaphor comprehension to working memory capacity (Prat et al., 2012), whereas others have argued that cognitive flexibility is required to select the common attributes of the ‘vehicle’ and the ‘target term’ and to shift between literal and metaphoric meanings (Mashal and Kasirer, 2011), with inhibition control being required to suppress irrelevant literal interpretations (Dietrich, 2004; Landa and Goldberg, 2005; Chiappe and Chiappe, 2007; Mashal and Kasirer, 2011; Beaty and Silvia, 2013; Iskandar, 2014). Thus, given that metaphor comprehension demands abstraction ability and is associated with executive functioning (Carriedo et al., 2016), then it can be postulated that executive dysfunction may explain difficulties in metaphor comprehension in ASD. Indeed, there is evidence showing that individuals with ASD demonstrate lower performance in metaphor comprehension due to their difficulties in executive functions (Russell, 1997; Mashal and Kasirer, 2011). Consistent with these findings, the results of the current study also show that children with ASD scored lower on conventional metaphor comprehension (but not on novel metaphor comprehension) than their age and language-matched TD peers, probably because children with ASD demonstrated poorer executive functioning, as evidenced by scoring less than their TD peers on executive function tests (i.e., fluency scores). It is important to note that the difficulties in EF observed in our ASD sample may explain the difficulties observed in conventional metaphor comprehension [as was also documented in Mashal and Kasirer’s (2011) study] but not seen while generating novel metaphors, as evidenced by higher scores of the ASD group than their TD peers in creative metaphor generation.

Some study limitations should be taken into account. Our measures of executive function were rather limited and may need to be expanded to include additional measures such as the assessment of planning, working memory, and inhibition. Indeed, there is evidence suggesting that people with ASD may experience difficulties in tasks that require response inhibition (e.g., Hill, 2004), planning an assignment (e.g., Olde Dubbelink and Geurts, 2017), or using working memory (e.g., Wang et al., 2017). Future studies will benefit from examining whether or not those executive functions are associated with verbal and figural creativity. The current study also did not test whether the children had similar drawing abilities, as different practice experience may have contributed to the findings (Julius and Adi-Japha, 2015, 2016; Julius et al., 2016).

### Implications

To conclude, the current study’s results indicate that children with ASD exhibit unique verbal creativity, as assessed by the novel metaphor generation task. They are also not impaired in figural creativity compared to their TD peers; in fact, the ASD group used more cross-category insertions in the non-existent object drawing task. Additionally, the two groups appear to rely on different abilities when generating novel metaphors: whereas children with TD appear to rely on their fluid intelligence to generate novel metaphors, children with ASD appear to use language-based executive functions. Such information may assist developing intervention programs aiming to enhance creative thinking tailored for each group’s capabilities.

Accordingly, this research suggests that verbal creativity (novel metaphor generation) and figural creativity (non-existent object drawing) are two separate abilities relying on different cognitive resources. The current findings suggest a unique creative cognition profile in ASD; future research should explore this possibility by assessing other verbal and figural abilities among this population.

## Data Availability Statement

The raw data supporting the conclusions of this article will be made available by the authors, without undue reservation.

## Ethics Statement

The studies involving human participants were reviewed and approved by Israeli Ministry of Education. Written informed consent to participate in this study was provided by the participants’ legal guardian/next of kin.

## Author Contributions

AK performed data curation and writing. EA-J performed conceptualization, analyzing, and participating in writing. NM performed conceptualization, methodology, participating in writing, and supervision. All authors contributed to the article and approved the submitted version.

## Conflict of Interest

The authors declare that the research was conducted in the absence of any commercial or financial relationships that could be construed as a potential conflict of interest.

## Appendix

Examples by ASD.

**Creative verbal responses (in Hebrew and literally translated to English):**

To feel embarrassed is to be in a cloud of humiliation.

להרגיש נבוך זה להיות על ענן השפלה

To understand is like suddenly putting on glasses.

להבין זה כמו לשים פתאום משקפיים

Feeling worthless is like a 10-penny coin.

להרגיש חסר ערך זה כמו מטבע של 10 אגורות

To love is to surf the rainbow.

לאהוב זה כמו לגלוש על קשת

**Drawings:**

Example of cross-category insertion by 12 years old child with ASD

(Crocodile head, snake neck and cockroach body).

Example of cross-category insertion by 11 years old child with ASD

(Head of a cow and body of a dog).

Example of cross-category insertion by 12 years old child with ASD

(House with hands and roof with fruit).

Example of cross-category insertion by 12 years old child with ASD

(Deer with ponytail and wings).

Example of same-category insertion by 12 years old child with ASD

(A lion with 3 heads and 3 tails).

Example of same-category insertion and deletion part of the drawing, by 12 years old child with ASD.

(A cat with 3 legs, without neck).
